# Risk sharing arrangements for pharmaceuticals: potential considerations and recommendations for European payers

**DOI:** 10.1186/1472-6963-10-153

**Published:** 2010-06-07

**Authors:** Jakub Adamski, Brian Godman, Gabriella Ofierska-Sujkowska, Bogusława Osińska, Harald Herholz, Kamila Wendykowska, Ott Laius, Saira Jan, Catherine Sermet, Corrine Zara, Marija Kalaba, Roland Gustafsson, Kristina Garuolienè, Alan Haycox, Silvio Garattini, Lars L Gustafsson

**Affiliations:** 1 Ministry of Health, Warsaw, Poland; 2 Division of Clinical Pharmacology, Department of Laboratory Medicine, Karolinska Institutet at Karolinska University Hospital Huddinge, Stockholm, Sweden; 3 Institute for Pharmacological Research 'Mario Negri', Milan, Italy; 4 Prescribing Research Group, University of Liverpool Management School, UK; 5 Agency for Health Technology Assessment, Warsaw, Poland; 6 Kassenärztliche Vereinigung Hessen, Frankfurt, Germany; 7 HTA Consulting, Krakow, Poland; 8 State Agency of Medicines, Tartu, Estonia; 9 Horizon Blue Cross Blue Shield of New Jersey, Newark, USA and Clinical Professor, Rutgers State University of New Jersey, USA; 10 IRDES (Institut de Recherche et de Documentation en Économie de la Santé), Paris, France; 11 Catalan Health Service, Barcelona, Spain; 12 Republic Institute for Health Insurance, Serbia; 13 Hospital Pharmacy, Karolinska University Hospital Solna, Stockholm, Sweden; 14 Faculty of Medicine, Vilnius, Lithuania

## Abstract

**Background:**

There has been an increase in 'risk sharing' schemes for pharmaceuticals between healthcare institutions and pharmaceutical companies in Europe in recent years as an additional approach to provide continued comprehensive and equitable healthcare. There is though confusion surrounding the terminology as well as concerns with existing schemes.

**Methods:**

Aliterature review was undertaken to identify existing schemes supplemented with additional internal documents or web-based references known to the authors. This was combined with the extensive knowledge of health authority personnel from 14 different countries and locations involved with these schemes.

**Results and discussion:**

A large number of 'risk sharing' schemes with pharmaceuticals are in existence incorporating both financial-based models and performance-based/outcomes-based models. In view of this, a new logical definition is proposed. This is "*risk sharing' schemes should be considered as agreements concluded by payers and pharmaceutical companies to diminish the impact on payers' budgets for new and existing schemes brought about by uncertainty and/or the need to work within finite budgets*". There are a number of concerns with existing schemes. These include potentially high administration costs, lack of transparency, conflicts of interest, and whether health authorities will end up funding an appreciable proportion of a new drug's development costs. In addition, there is a paucity of published evaluations of existing schemes with pharmaceuticals.

**Conclusion:**

We believe there are only a limited number of situations where 'risk sharing' schemes should be considered as well as factors that should be considered by payers in advance of implementation. This includes their objective, appropriateness, the availability of competent staff to fully evaluate proposed schemes as well as access to IT support. This also includes whether systematic evaluations have been built into proposed schemes.

## Background

Expenditure on pharmaceuticals is rising more rapidly than other components of health care with costs of cancer products growing at 21% per annum in recent years [1-5]. This will continue with ongoing demographic changes, instigation of stricter clinical targets and the continued launch of new expensive drugs [5-8]. Increasingly, though there are concerns with the level of health gain of new drugs with a median of just 0.097 Quality Adjusted Life Years (QALYs) versus current standards in an analysis of 281 recent submissions by pharmaceutical companies to the Scottish Medicines Consortium [9]. This compares with the public perception that many new drugs are seen as breakthroughs [10].

European governments and health authorities have introduced successive reforms and initiatives to address these challenging resource issues including funding new premium priced innovative drugs. These include measures to engineer low prices for generics and originators as well as interchangeable brands within a class [2,11-16]. They also include steps to improve transparency in classifying new drugs as innovative, linking the perceived degree of innovation of new products to reimbursed prices, and limiting payer exposure to new expensive drugs given their potential significant budget impact [11,13,17-23]. Not surprisingly, optimising the use of new expensive technologies is seen as the major challenge faced by health services in Europe as they strive to continue providing comprehensive and equitable healthcare [6].

Current schemes to limit the growth in pharmaceutical expenditure, ensure health gain is maximised within finite budgets, or both including limiting 'off label' prescribing and prescribing outside identified sub-populations where the value of the technology is greatest. They also include price-volume agreements (PVAs), patient access schemes where typically drugs are provided free for a period of time, 'coverage with evidence development' schemes as well as payment by result schemes based on outcomes achieved in practice. These latter schemes are also known as performance-based contracts, efficiency stipulation schemes or effectiveness guarantee schemes [13,19-22,24-35]. All of these schemes have been included within the general term 'risk sharing schemes' either by payers or pharmaceutical companies.

The number and range of risk sharing schemes have grown in recent years no doubt enhanced by patient and physician pressure on governments to accelerate access to new and more costly medicines despite often significant uncertainty surrounding their likely health benefit [10,36]. This may well be exacerbated by pharmaceutical companies keen to address lost revenues from patent expires, which have recently been estimated at over US$100 bn per year over the next four years [37]. Payers though need to consider the opportunity costs of risk sharing schemes if available resources are not used wisely [36,38].

Further supply side and demand side initiatives are essential if Europe is to maintain the ideals of socially funded and equitable healthcare. This could mean further expansion of risk sharing schemes. There have been a number of papers published recently that have comprehensively reviewed existing and historic risk sharing schemes offering suggestions for the future [5,10,20,34,36,39]. However, there is still considerable confusion surrounding the terminology, which urgently needs to be addressed. There are also concerns among health authority personnel with the level of administrative intensity associated with some of the current schemes and that they could end up contributing substantially to the development costs of new molecules [5,40,41]. Certainly in the past, a great deal of the risk associated with outcomes of coverage decisions have been borne by health authorities and insurance companies [34]. This is starting to change given the number of new expensive technologies being launched coupled with their budget impact [6,23,34]. These challenges are magnified by the lack of scientific studies evaluating the implementation and outcome of many existing schemes for pharmaceuticals in terms of their overall costs and benefits [10,34]. This has not been the case with non-pharmacological technologies where 17 policy outcomes have been derived to date from 32 principally non-drug technologies, with in approximately 60% of cases the coverage decision consistent with the outcomes of the study conducted as part of the schemes [34].

Benefits of risk sharing schemes include enhancing health gain within available resources as well as addressing issues such as the safety of new products in practice [10]. These benefits though have to be balanced against concerns for patient care if new drugs are launched too early with considerable uncertainty regarding their safety as well as potentially paying for cost-ineffective technologies (type I error) [36].

We believe these concerns need to be discussed and debated before there is a continuing surge in 'risk sharing' schemes. This paper aims to stimulate this debate by:

• Proposing logical definitions which can act as a reference for the future

• Documenting exemplars from the published literature to illustrate proposed definitions supplemented with additional examples from the co-authors

• Documenting the published impact, concerns and outcomes from current schemes from a payer perspective

• Summarising considerations that should be borne in mind by payers when contemplating future schemes

• Proposing guidance on key criteria for considering future risk sharing schemes again from a payer perspective

### Methodology

A literature review was undertaken by one of the authors (BG) in PubMed, MEDLINE and EMBASE between 2000 and February 2010 using key words. These were 'conditional coverage', 'conditional reimbursement', 'risk sharing', 'coverage with evidence', 'price volume agreements', 'value-based pricing, pharmaceuticals', 'no cure no pay', 'pay back schemes', 'health impact guarantee' and 'outcome guarantee'. This was supplemented by unpublished or "grey literature" references known to the 16 co-authors from European countries and locations involved with considering, evaluating and/or implementing such schemes. The focus is principally on Europe although other schemes relating to pharmaceuticals are also documented for Australia, Canada and the US to provide additional examples.

Only papers documenting the nature and content of actual schemes were considered for possible inclusion in this paper. There was no attempt to assess the quality of published papers using established criteria such as the Cochrane Collaboration criteria [42]. This was because this is a 'correspondence' article undertaken by health authority and health insurance personnel evaluating and implementing such schemes rather than a comprehensive review of all risk sharing schemes undertaken as such reviews have recently been published [10,20,34].

The schemes were subsequently collated under the proposed definitions to provide guidance to health authority and health insurance personnel as these are the principal intended audience for this paper. Gaps in the knowledge base have been identified with suggestions for the future.

Where possible, Euros are quoted; however, some figures are quoted in GB£s and US $ (€1 = US$1.36, GB£0.90, SEK9.70 - 3 March 2010).

### Definitions

We propose 'risk sharing' schemes for pharmaceuticals should be considered as agreements concluded by payers and pharmaceutical companies to diminish the impact on the payer's budget of new and existing medicines brought about by either the uncertainty of the value of the medicine and/or the need to work within finite budgets. In practice, the agreement lies in setting the scope and realizing the mutual obligations amongst both payers and pharmaceutical companies depending on the occurrence of an agreed condition - the "risk". The "risk" varies by situation, and can include pharmaceutical expenditure higher than agreed thresholds or health gain from a new product lower in practice reducing its value.

We recognise our definition is different to the definition recently proposed by Towse and Garrison - namely these are '*agreements between a payer and a pharmaceutical company where the price level and/or revenue received is related to the future performance of the product in either a research or real-world environment' *[36].However our efforts have been directed towards finding a definition coherently overarching the many agreements introduced in previous years as well as one acceptable to health authority and health insurance personnel. Consequently, we believe it is essential to create a definition according to rules of a logical division. These are firstly that there is only one basis of the division. Secondly, every subject belongs to only one group, where there may be sub-categories of equal rank. Thirdly, every example form the superior group, i.e. risk-sharing schemes, has to fall into either category, i.e. either financial based or performance based.

All the schemes discussed and proposed should have a common denominator that is the "risk shared by the payer and the pharmaceutical company". What differentiates the various schemes is the nature of the risk, i.e. "a probable situation in future". Based on our proposed definition, the many existing 'risk-sharing' schemes can be subdivided into either financial/financial-based models or outcome/performance-based models. This is broader than the definition proposed by Towse and Garrison, which just covers performance-based schemes. However, it is a workable definition from a payer perspective.

Even this division, despite being based on common naming, may be misleading though because it suggests that outcome or performance-based models have little to do with expenditure. In fact, the opposite can be true with price-volume agreements and other financial schemes often having little to do with patient outcomes, concentrating more on keeping expenditure within agreed limits and/or enhancing the value proposition of the new product. Price capping schemes do though bring in an outcome element.

The various examples of risk sharing scheme included within the proposed definition are:

• Financial based schemes:

∘ Price-volume agreements (PVAs)/budget impact schemes. These focus on controlling financial expenditure with pharmaceutical companies refunding over budget situations

∘ Patient access schemes. These typically involve either free drug or discounts for an agreed period to enhance the value of new medicines and improve the possibility of their funding/reimbursement. Patient access schemes also include price-capping schemes, which focus on controlling the financial impact but from an individual patient perspective. Typically drugs are provided free once patients have exceeded an agreed utilisation limit to again enhance reimbursement/funding within finite resources

∘ Performance based/outcome-based models. These can include schemes whereby companies refund agreed monies or provide free drug if the desired outcomes are not reached. Alternatively, a price reduction if the new drug fails to deliver the desired health gain in practice. In reality, the latter is likely to lead to price shifting from payers to manufacturers as new drugs may well not be able to fully reproduce the desired benefits once prescribed in a wider population than those in the clinical trials, i.e. the net monetary benefit is lower in reality

### Existing schemes

As discussed, we believe all existing schemes can be collated into two groups; namely financial/financial-based models or performance-based/outcomes-based models.

Examples of financial-based models include price-volume agreements (PVA) - sometimes referred to as budget impact schemes - and patient access schemes including price capping schemes are included in Table 1[12,13,28,36,43-53], Table 2[20,29,33,34,54-57] and Table 3[5,19-21,24,31,32,34,58-68]. This is not a complete list but aims to give a number of examples.

**Table 1:** Examples of Price Volume Agreements (PVAs)/budget impact schemes in Australia and Europe

Country	Examples
Australia	PVAs have been in place for a number of years in Australia with price reductions if sales exclude pre-agreed volumes as well as rebate arrangements if costs exceed a subsidised cap or threshold More than 50 such 'risk sharing' arrangements have been instigated to date

Belgium	A payback mechanism has existed since 2002, with the regulation written into the legislation Originally, each pharmaceutical company paid an advanced percentage of their sales based on anticipated expenditure and the previous year's sales Refunds were subsequently adjusted based on real expenditure, with pharmaceutical companies and insurers making up part (65%) of any realized excess In 2006, the payback system for exceeding expenditure was replaced by a "Provision fund" system where a fund of € 100 mn was created through advances paid by pharmaceutical companies, with companies covering 75% of any over run Additional refunds are requested during the financial year if €100 mn is insufficient

Estonia	PVAs are administered by the Ministry of Social Affairs. They are valid for a minimum of one year, and are obligatory for all pharmaceuticals in the reimbursement system (positive list) else products will be 100% co-pay Pharmaceutical companies are required to state the rationale behind requested prices and volumes. If the Ministry still feels suggested prices are too high, products are not reimbursed and/or delisted If agreed volumes are exceeded, negotiations take place between the Ministry of Social Affairs and the pharmaceutical company to determine the rationale and course of action. Agreed actions may include lowering reimbursed prices No action is a possibility if excessive volumes could not have been foreseen by Ministry personnel and they now believe higher volumes should be funded

France	Two schemes exist in France. These include a payback mechanism for excessive sales by therapeutic class and are based on pharmaceutical company’s agreed turnover with annual financial adjustments. They also include regular price reviews based on the average daily costs, the average dose or the total number of units established at the time of reimbursement. Payback mechanisms per class are not the same each year Previous payback schemes have included 65% covered by all companies marketing the drugs in the class and 35% by companies whose sales exceed agreed thresholds New drugs with an ASMR I are exempt from such agreements for 36 months after launch, ASMR II for 24 months, ASMR III for 24 months at a level of 50% and ASMR IV at a level of 25% for 24 months. Generic are totally exempt Since 2008 orphan drugs are no longer automatically exempt with total sales of €815 mn in 2008 and growing rapidly, and are now subject to specific agreements with payback mechanisms for sales above agreed levels. There were two schemes in 2008; the first involved Naglazyme^® ^- for the treatment for mucopolysaccharide type VI disease - and the second involved Soliris^® ^- for the treatment of paroxysmal nocturnal haemoglobinuria In 2004, total rebates amounted to €670 mn - some 3% of total pharmaceutical expenditure. Rebates were €260 mn in 2008.

Germany	Several rebate contracts and other mechanisms exist between the Sickness Funds and pharmaceutical companies to accelerate access and/or enhance market penetration of certain drugs where there are concerns with their value. This includes the so called target agreements Current schemes include the insulin analogues, olanzapine, risperidone, clopidogrel, zolendronate (Aclasta^®^), mycophenolic acid (Myfortic^®^), everolimus(Certican^®^), and cyclosporine (Sandimmun^®^) There is though typically secrecy between the Sickness Funds and the Pharmaceutical companies concerning crucial issues such as the actual scope, measurements and time frames, which can cause problems for the State Physician Associations

Hungary	A general payback scheme has been in operation since 2003 based on individual products as well as total pharmaceutical expenditure Since January 2007, pricing criteria for pharmaceuticals is regulated by law with only limited exceptions Under the scheme, companies must pay the Ministry 12% of their total reimbursed sales each year. If this is insufficient to cover the agreed budget overspend, companies must pay additional monies according to an agreed formula. For the first 9% of any overspend for a given class, the social health insurance and pharmaceutical companies share the cost of the additional overspend, with pharmaceutical companies paying a greater percentage. If the overspend exceeds 9% of the agreed budget, pharmaceutical companies cover all the additional costs themselves Rebates are based on the individual company's share of reimbursed sales for the class. Alongside this, there is also a payback scheme for certain target pharmaceuticals. Depending on the individual contract, there are also yearly (or monthly) refunds based on reimbursed sales. There is a sliding scale, with 100% rebates for any over budget expenditure above agreed limits for the product The payback in 2006 was 22.5 billion HUF (€90 mn - 5.69% of the budget)

Italy	Compensation schemes exist where there is excessive prescribing and costs of pharmaceuticals above agreed limits. Current limits for pharmaceutical expenditure in primary care are 14% of total NHS expenditure and 2.4% of total NHS expenditure in hospital care Rebates amounted to €773 mn in 2005

Lithuania	PVA schemes are administered by the State Patient Fund under the Ministry of Health From 2008, such schemes are obligatory for all new pharmaceuticals that will increase the Statutory Health Insurance drug budget compared with current treatment approaches for the target patient population. Once instigated, PVA scheme are currently valid for a minimum of three years If agreed sales volume (expenditure) exceeds the agreed target, pharmaceutical companies must refund all the difference

Portugal	Since 1997, there have been 4 rebate agreements to limit reimbursed pharmaceutical expenditure signed between the Ministry of Health and the Portuguese Pharmaceutical Industry Association (first time the industry association has been involved) The agreement, signed in February 2006, included both ambulatory care and in-patient hospital expenditure following the instigation of formal Pricing and Reimbursement for hospital products (prior to this just ambulatory care). Hospital products were included for the first time as many new expensive medicines are being launched in hospital The agreement ran from 2006 to 2009. Under this agreement, no growth was permitted in ambulatory care pharmaceutical expenditure in 2006 vs. 2005, with only a nominal growth rate in 2007 vs.2006. The rate depended on the actual increase in GDP for that year. The growth rate for in-patient drugs was limited to 4% in 2006 vs. 2005. There are exceptions though for new in-patient drugs for cancer and HIV as well as potentially single agents in other disease areas depending on the circumstances. Before this agreement, pharmaceutical companies negotiated directly with hospitals In case of any over budget, companies provide refunds equal to 69.6% of the increase up to maximum of € 35 mn in 2006 and € 45 mn in 2007. Part of this refund is diverted to a special fund supporting research

**Table 2:** Examples of patient access schemes in Australia and Europe including free drug or discounts

Country	Examples
Australia	There are pricing arrangements for Section 100 drugs (restricted supply of specialist drugs to hospitals or other similar facilities) whereby companies typically provide free drugs to lower the cost per unit; alternatively provide an agreed percentage discount to Medicare Australia Examples include Abacavir - the Pharmaceutical Benefit Scheme would only pay for 2 packs for every 3 supplied, Cirone progesterone gel - Listing was achieved with the help of a 49.5% discount, and Deferasirox - a 20% discount was applied to achieve reimbursement

Serbia	Patient access schemes were initiated in 2008 to enhance the value of three cancer drugs, namely bevacizumab, cetuximab and mabCampath For these medicines to be included (reimbursed) in the positive list covered by mandatory health insurance, specific agreements were established between the Serbian Health Fund and the manufacturers Under the terms, the manufacturers agreed to rebate of 25% of the reimbursed costs in 2008; this was reduced to 11% in 2009. One cancer treatment was excluded as the manufacturer did not want to enter into the scheme

UK (England, Wales)	NICE (National Institute for Health and Clinical Excellence) has recently entered into a number of patient access programmes to enhance the value of new medicines Examples include cetuximab as first line treatment of metastatic colorectal cancer. Under this scheme, Cetuximab will be rebated as free stock (1 vial per 8 utilised) when used in combination with FOLFOX. Alternative methods will be found if 'free stock' is not suitable. In addition, patient registration is essential to ensure scheme integrity and NICE usage criteria are followed. The Trust pharmacy will carry out a monthly/quarterly audit of usage to make claims from the manufacturer, with free stock delivered directly to the Trust Other examples include Sunitinib for patients with metastatic renal cell carcinoma. Under this scheme, the first treatment cycle (6-weeks costing an average of GB£3139/patient) is provided free via a patient access programme. Subsequent cycles are funded by the NHS until disease progression. The Department of Health considered the scheme did not constitute an excessive administration burden on the NHS Sorafenib for metastatic renal cell carcinoma is another example. Under this scheme, the first pack (200 mg × 112 tablets) is provided free by the manufacturer under the agreed patient access programme. This equates to £2980.47 p excluding VAT

UK (Scotland)	A Patient Access Scheme Assessment Group (PASAG) has recently been established under NHS National Services Scotland reviewing and advising NHS Scotland on the feasibility of proposed schemes for implementation. PASAG operates separately from the Scottish Medicines Consortium (SMC) in order to maintain the integrity and independence of SMC's assessment process. The first scheme was accepted in November 2009 Schemes accepted by NICE may be implemented in Scotland if the medicine was previously accepted by SMC prior to November 2009 or if the medicine has been assessed via the NICE multiple technology appraisal process and the advice has been accepted by NHS Scotland Examples include cetuximab where the manufacturer estimated that the budget impact in Scotland for suitable patients would increase from £1.8 mn in year 5 to £2.5 mn if no patient access scheme was in place

**Table 3:** Examples of patient access schemes involving price caps in operation in Europe and US

Country	Examples
Italy	Bevacizumab for the management of approved cancers cannot exceed €25,941 per year

Sweden	Stockholm County Council initially signed an agreement in April 2008 lasting until end December 2009 whereby if patients with advanced cancer exceeded an accumulated dose of 10,000 mg of bevacizumab, the additional costs would be fully covered by the Company The scheme has now been extended into 2010 Other regions in Sweden have also been offered similar schemes

UK - England, Wales	Schemes include the Ranibizumab Reimbursement Scheme. Under the scheme, the first 14 injections in the eye for the management of wet age-related macular degeneration (AMD) are paid for by the national health service with patients demonstrating an 'adequate response' to therapy to continue with treatment. The drug costs of any subsequent ranibizumab injections will be reimbursed by the company (Novartis) either as free drug or as a credit note Other schemes include Lenalidomibe for patients with multiple myeloma who have received prior therapy. This scheme was approved to enhance the cost effectiveness of lenalidomibe. Under this scheme, the manufacturer pays the cost of the drug if more than 26 cycles are needed for any patient (approximately 2000 patients in the UK) - equating to any patient needing more than 2 years of therapy Ustekinumab for moderate to severe psoriasis is another example. Under this scheme two 45 mg vials (90 mg) are provided for people who weigh more than 100 kg at the same cost as a single vial in the form of free drug

UK - Scotland	Schemes incude'Ranibizumab Reimbursement Scheme - as above In addition Ustekinumab - as above. SMC estimates that this patient access scheme will minimise the additional budget impact so long as prescribing is in line with the manufacturer's proposed positioning

US	Programmes were introduced by Genentech in the US in 2006 to cap the total cost of bevacizumab at $55,000 per year for patients below an income of $75,000 per year. Costs are a particular issue especially for patients with breast cancer, as well as earlier stages of lung and colon cancer, with the scheme resulting in a 50% reduction or more in costs for one year of treatment. The company believed this would help address public concern over the rising prices for cancer drugs ImClone Systems and Bristol-Myers Squibb announced in 2006 that lower-income patients who reached a price cap of approximately US$10,000 monthly for cetuximab could receive additional supplies at no extra cost, or at a large discount. This administered through an independent charitable programme Amgen in 2006 introduced the Oncology Assistance (AOA) programme to provide financial assistance for patients prescribed panitumumab for the treatment of metastatic colorectal cancer when co-payments reached 5% of patient's adjusted gross income. This administered via a Foundation Pfizer recently launched the MAINTAIN scheme running from 1 July 2009 to 31 December 2009. Under the scheme, Pfizer will help patients who have recently made redundant to continue obtaining their medicines There are also a number of assistance programmes for patients with HIV both for antiviral drugs to treat the disease as well as side-effects of therapy. Eligible patients include those with low income not covered by existing programmes including Medicare Part D In addition, Managed Care Organisations have also instigated maximum dose policies with manufacturers to reduce their exposure, e.g. United Healthcare

Any further discussion on specific patient access programmes in the US is outside the scope of this paper.

Table 4 gives details of performance-based or outcome-based models in Canada, Europe and USA [10,19,20,25-27,34,40,51,69-77]. Again, this is not an exhaustive list but aims at giving a number of examples.

**Table 4:** Examples of performance-based or outcome-based models in Canada, Europe and US

Country	Examples
Canada	Sandoz Canada promised to reimburse individuals, hospitals and government drug plans where patients with treatment-resistant schizophrenia discontinued clozapine within six months. This was initiated to address acquisition cost concerns versus typical anti-psychotics among the Provinces Merck-Frost offered to reimburse provincial governments the full cost if patients prescribed finasteride subsequently required surgery for benign prostatic hyperplasia after one full year of medical therapy Sanofi-Aventis agreed to reimburse the cost of docetaxel to provincial payers if an agreed responder level was not reached in patients with cancer due to concerns about its efficacy and costs. The programme lasted six months facilitating formulary listing

Denmark	A population based 'no cure, no pay' strategy for valsartan to lower BP was initiated to enhance market share Money back initiative for nicotine chewing gum if patients do not like the taste of any of the four flavours on offer 'No play; no pay' schemes for drugs for erectile dysfunction

Italy	i) *CRONOS scheme for Alzheimer drugs* Initially the acetyl cholinesterase inhibitors were 'C' classification in Italy, i.e. 100% co-payment However, under the CRONOS scheme, companies initially provided acetyl cholinesterase inhibitors such as donepezil free of charge to specialist clinics for the first four months of treatment The NHS subsequently covered the drug costs in responders, with patient outcomes recorded This observational study, which demonstrated health gain in patients with mild to moderate AD, resulted in the NHS subsequently funding these drugs ('A' classification) provided patients were treated in specialist outpatients. However, there were no quality checks on the completed forms *ii) Ongoing registries to monitor prescribing and therapeutic value in practice* A number of registers have been initiated in Italy to monitor prescribing against licensed indications as well as monitor their therapeutic value in practice to guide future management and reimbursed prices This includes a number of premium priced drugs such as cetuximab, ibritumonab tiuxetan, lenalidormibe, nelarabine, palifermin, temporfin and trastuzumumab for adjuvant use Overall over 43,000 Italian patients have been included in the registries for new cancer medicines (up to Oct 2009)

UK - England and Wales	i) *Beta interferon for multiple sclerosis (MS)* NICE in its initial appraisal rejected funding for the β interferons in the treatment of MS on clinical and cost-effectiveness grounds with a calculated a cost/QALY of £42,000 to £98,000 over 20 years rising to a maximum of £780,000/QALY over 5-years Following external pressure, the government in 2002 established a scheme with the four manufacturers whereby a cohort of approximately 10,000 patients would be followed for over 10 years with the cost of drugs reduced or refunds given if the cost/QALY over an envisaged 20-year horizon was over £35,000/QALY, i.e. fund a maximum value of £35,000/QALY or less Patients would be followed using the Kurtzke Expanded Disability Status Scale (EDSS), which was the same outcome measure used in the trials However, the scheme has been heavily criticised as unscientific and impractical An initial assessment was published in 2009 reviewing patients who started treatment from May 2002 to April 2005. This highlighted important methodological issues with this study and the need for longer term follow-up before securing meaningful results *ii) Bortezomib for the treatment of first relapse of multiple myeloma* This scheme is based on a 50% reduction in serum paraprotein levels (M-protein) by the fourth cycle. The NHS will continue funding treatment in responders, with the cost/QALY reduced from £38,000/QALY to a more acceptable £20,700/QALY, with manufacturers refunding the cost of the drug if a 50% reduction was not achieved. This is usually in the form of free drug, which is seen as easier to implement In addition, prices remain at the launch price despite up to a 60% discount in reality, which is important with the UK often used as a reference price country However there have been concerns whether M-protein is a good surrogate for life expectancy. Alongside this, 10 to 15% of patients do not have measurable serum M-protein levels *iii) Atorvastatin for CHD prevention* The pharmaceutical company agreed to fund the health authority for wasted resources if atorvastatin failed to reduce LDL-C levels to agreed targets when patients have been properly titrated No refunds were given in reality as all properly titrated patients reached target lipid levels helped by the recruitment of two nurses. The nurses worked with GPs and practice nurses aiding issues such as concordance, although a 20% adjustment was included in the outcome guarantee model GP and patient participation was helped by CHD being a high priority disease area in the UK with national initiatives to improve care However, there were problems with the scheme once generic simvastatin became available and lipid level targets were lowered in the UK *iv) Pharmaceutical Price Regulation Scheme (PPRS) proposals* Under the proposed flexible Value-Based Pricing (VBP) in the new PPRS scheme, pharmaceutical companies will be able to establish the initial launch price for their new products Reimbursed prices will either fall or rise as new evidence of effectiveness and safety becomes available, or when new indications are added changing the overall value, following further appraisals by NICE

UK - Scotland	Beta interferon for multiple sclerosis (as for England) PPRS proposals apply to Scotland although the implementation of flexible pricing is still being discussed

US	• Patients and insurers were refunded if simvastatin failed to lower LDL to target levels (up to 6 months) • CIGNA agreed with pharmaceutical companies that they would reimburse the cost of treating a heart attack if this occurred whilst patients were being treated with lipid lowering drugs • 'No cure, no pay' for valsartan and valsartan hydrochlorothiazide to patients and insures as part of a 'take action for healthy blood pressure' initiative. In addition a number of compliance enhancing initiatives were simultaneously launched by the company to help achieve BP target goals including the option to purchase a blood pressure monitoring device at significantly reduced costs • Merck agreed to refund the cost of the drug for patients whose symptoms of benign prostatic hyperplasia did not improve within six months or who required surgery within two years assessed based on pre-determined criteria

Others (no particular country)	• 'No baby - no pay' option for fertility treatments funded through national health services

In addition to these schemes, the Italian Medicines Agency (AIFA) has recently instigated two different approaches to accelerate reimbursement for new drugs especially where there is limited data available at launch. These include variations on patient access schemes as well as performance-based/outcome contracts [5,34,51]. The AIFA Oncologic Working Group suggested two risk sharing arrangements for new anti-cancer medicines to enhance their reimbursement potential based on:

• Epidemiological data for the disease

• The possibility to clearly define a subset of the population responsive to the treatment

• Results from the submitted clinical trials

Six products were included in the scheme with a further product added in 2009 [5,34,51]. These were:

• Bevacizumab - Metastatic carcinoma of the colon or rectum, breast cancer, NSCLC and advanced and/or metastatic renal cell cancer

• Dasatinib - Chronic myeloid leukaemia and acute lymphoblastic leukaemia

• Erlotinib - NSCLC after failure of at least one prior chemotherapy regimen

• Nilotinib - Chronic myeloid leukaemia

• Lapatinib - HER2+ breast cancer patients

• Sorafenib - Second line treatment for renal cell carcinoma and hepatocellular carcinoma

• Sunitinib - Metastatic renal cell carcinoma (first and second line treatment)

Inclusion in either risk sharing scheme depended on the characteristics of the drug and the tumour type and included:

• Erlotinib, sunitinib and sorafenib - provided at 50% discount from current prices for an agreed number of cycles. In the case of erlotinib this is 2 months; 12 weeks for sunitinib and sorafenib. As a result, with erlotinib part funding the 50% of patients who would be expected to have disease progression at or before 8 weeks of treatment

• Dasatinib and lapatinib - the Italian Health Service fully covers the cost of drugs for the responders following assessment; manufacturers refunding the costs in the case of disease progression

Four new drugs are also currently contained within the Italian conditional reimbursement scheme [34,51]. This is similar to the US Centre for Medicare and Medicaid conditional coverage scheme under which conditions are set for the continued reimbursement of new technologies [5,34,39]. The current scheme in Italy covers ivabradine for chronic angina pectoris, as well as exenatide, sitagliptin and vildagliptin for patients with Type 2 diabetes resistant to current oral treatments [34,51]. Under this scheme, AIFA fully reimburses the new drugs until further re-evaluation of their actual level of innovation. The main objectives being to evaluate utilisation in routine clinical practice, collect epidemiologic data as well as obtain data on the effectiveness and safety of the new drugs in practice. The measures used to assess effectiveness of the new drugs for diabetes are HbA1c levels. It is the number of angina episodes for new drugs for chronic angina pectoris. By the end of 2008, over 17,000 patients had been enrolled with 7% withdrawals due to therapeutic failure [51]. More recent schemes for new anti-diabetic drugs in Italy have concentrated on co-utilisation with either metformin or sulphonyl ureas. This is because these new drugs should only be prescribed if HbA1c levels are not controlled with existing regimens, or there are unacceptable side-effects with existing drugs at prescribed doses.

### Concerns with current schemes

There are already a considerable number of 'risk sharing' schemes in operation globally. However, there are concerns with existing schemes that need be addressed before they should become a routine part of future reimbursement or contracting negotiations especially given the suspicion among payers that a number of proposed schemes are extensions of pharmaceutical company marketing activities.

Whilst price-volume agreements (PVA) shift cost considerations from the payer to the pharmaceutical company, which is important especially if there are concerns that new medicines will be prescribed in a wider population than envisaged, there are concerns that the patients initially prescribed the drug will not always be those most likely to gain the greatest benefit [10,36]. The growing use of health informatics can help here. In addition, PVA schemes may not always consider issues such as compliance, which is a growing issue with the recent study by Cramer and colleagues showing that only 59% of patients take their cardiovascular and antidiabetic medication for more than 80% of days on therapy each year [78]. There are also concerns that pharmaceutical companies may additionally benefit from early access of new technologies with as yet unproven efficacy and safety even within such schemes unless the value of the new drugs has been critically evaluated by trained professionals. However, it is recognised PVA schemes may be the optimal method to finance new medicines especially where there are currently limited demand side measures to control off-label prescribing or prescribing in patient populations where the new medicine is less cost-effective [36,79].

Physician Association concerns in Germany with discount and rebate initiatives between Pharmaceutical Companies and the Sickness Funds in Germany include a lack of transparency. This can create problems for the Physician Associations in calculating and monitoring drug budgets as well as providing prescribing guidance to meet their responsibility to monitor and manage drug budgets and advise physicians when their allocated budgets are likely to be breached. This is especially important in Germany as physicians can be fined for being over budget [80]. Physician Association advice can be different to the advice given by Sickness Fund personnel to ambulatory care physicians to help improve the quality and efficiency of their prescribing especially if the value of discussed drugs is altered by discounts given by pharmaceutical companies. Matters can be further complicated when rebates and discounts are given for one drug on condition that there will be a predefined increase in utilisation and expenditure of a second drug.

This is different to the US where combined or bundling of rebates is now generally discouraged by Managed Care Organisations (MCOs) as it impedes transparency. This is particularly important where rebates are passed onto patients. As a result, contracts are increasingly focusing on individual drugs. Proposals for formulary inclusion are also increasingly being considered for drugs where there is support to improve adherence and compliance given concerns that poor compliance could severely compromise effectiveness potentially leading to increased medical and/or drug expenditure in practice to produce the desired clinical outcomes [78,81-83]. MCOs such as Horizon Blue Cross Blue Shield of New Jersey are conducting research to improve the understanding of barriers to enhance compliance, with the findings likely to be incorporated into future initiatives including formulary listing considerations. Having said this, the first consideration for formulary inclusion of new medicines should always be their comparative efficacy and safety versus current formulary drugs. Only when two medicines are seen as essentially similar will the inclusion of schemes aimed at enhancing adherence and compliance be considered for potential formulary listing. Alongside this, 'price protection' clauses are also now being written into some contracts where manufacturers have sought price increases in the past.

Concerns with patient access schemes involving free drug or discounts include opportunity costs, even if this reduces the overall cost/QALY for the new product, as this could still result in significant additional cost for the payers. Alongside this, the administrative burden associated with additional patient monitoring as well as collating and submitting information are also concerns. Concerns with price capping schemes again include the administrative burden such as proof that only appropriate patients have been incorporated into the scheme and that they have reached the agreed limit. This is especially important when the number of patients where these will be applicable in reality is low. For example, there is only one eligible patient in Serbia that would currently qualify under the price capping scheme for bevacizumab in operation in either Italy or Sweden (Table 3). Alongside this, obtaining replacement stock or credit can be a lengthy process especially if manufacturers operate via wholesalers. Such processes may also impact on the accuracy of local medicine utilisation systems making future comparisons difficult. Lastly, pharmaceutical companies may wish to help with data collection and verification, which has to be denied if there are issues with patient integrity.

There are a number of objections with performance-based or outcomes-based models. These include [4,9,10,20,34,36,41,81,84-90]:

• Whether the objective of the scheme is fully explicit

• Whether the level of evidence is sufficient to make robust decisions initially. This includes whether there is a sufficiently strong correlation between any surrogate measures included in the phase III trials and the postulated health gain. This also includes concerns if meta analyses have been undertaken with only indirect comparisons. Limited outcome data also makes it difficult to undertake any sub-group analysis to ascertain potential patient populations where the value of new drugs will be greatest

• Who will fund additional evidence generation given the high acquisition costs of most new technologies and concerns that considerable expenditure will be spent on marketing activities to promote new drugs rather than spent on further clinical trials

• Who will fund drug provision if rebated drugs go out of date before suitable patients can be found

• The reliability of registry and other data generation schemes unless comparable control groups. Concerns can be diminished if subsequent studies are undertaken by independent organisations

• Accelerating the uptake of new drugs in practice, i.e. including patients in registry studies or provisional reimbursement schemes may accelerate their uptake in practice. The utilisation of new drugs may also be greater if companies provide additional support such as nurses to help with case finding and monitoring. This may be balanced though through reducing 'off label' use if physicians know prescribed indications are being monitored.

• The length of follow up - especially if this is long (β interferon scheme in the UK - below)

• The general administrative burden for all key stakeholders. This also includes the costs associated with the instigation of additional evaluation units such as those proposed by NICE in England. The PASAG (Patient Access Scheme Assessment Group) is already established in Scotland to help evaluate proposed risk sharing schemes; similarly the Patient Access Scheme Liaison Unit within NICE. Both units are already adding to the administrative burden of current risk sharing schemes

• Compliance - especially for long term chronic conditions. This issue must be fully addressed where pertinent else all key stakeholder groups will lose out

• Pricing, i.e. pharmaceutical companies may be tempted to initially over price their new drug in expectation of price cuts/cost shifting downstream as the evidence base grows

On the other hand, pay-for-performance schemes do encourage pharmaceutical companies to develop biomarkers or other methods that help target patient populations where health gain and hence value is greatest. This is especially important for new cancer drugs given the limited aggregate health gain of most new drugs including those for cancer and their appreciable cost per patient [9].

There have been a number of concerns with the β interferon scheme for multiple sclerosis in the UK, which need to be considered by payers when evaluating future schemes [25,40,91-93]. These include:

• **The model**

• Flaws in the actual model due to difficulties in fully mapping out the quality of life and natural history of MS to the trial outcomes, which were based on changes in EDSS scores (Expanded Disability Status Score)

• Concerns that the model was heavily influenced by assumptions about future discounting and did not account for example for the cost of azathioprine

• The model did not appear to fully account for patients discontinuing treatment early because of side-effects

• **The length of follow-up**

• Concerns that within ten years the β interferons and glatiramer acetate may have been supplanted by newer drugs reducing the whole rationale behind the scheme

• **Funding and administration support**

• Primary Care Trusts generally did not receive any additional funding to cover the cost of these drugs

• Hospitals also did not receive additional funding for more extensive follow-up consultations and for completing the necessary forms reducing their involvement in practice

• Concerns generally with the necessary infrastructure required including specialist nurses as well as concerns where the costs of the additional administrative burden would come from

The administrative burden, lack of communication, and concerns with passing on savings have all been highlighted as key issues with current schemes for cancer drugs in the UK [41]. Recent research among hospitals yielded [41]:

• 73% of hospitals reported they did not have the capacity to manage current schemes as these typically required additional staff to manage, co-ordinate and track them. This is especially the case if hospital personnel have to spend valuable time manually tracking patients, retrospectively adjusting stock control systems and ensuring necessary financial systems are in place to fully realise any savings

• A need for greater flexibility around the time limits for processing claims

• A need for good communication between key stakeholder groups, e.g. in the case of bortezomib every missed claim results in a loss of GB£12,000

• The need to ensure savings are passed back to the payers - this is not happening in 47% of hospitals

These findings again highlight concerns with existing schemes that need to be adequately addressed in the future before risk sharing schemes become a routine part of future reimbursement considerations.

## Conclusions and future proposals

We believe there are only a limited number of situations where 'risk sharing' schemes should be considered in the future, as well as key issues that need to be considered by payers before entering into future 'risk sharing' arrangements. These are summarised below and have arisen due to the increasing launch of new expensive technologies putting considerable strain on European healthcare systems. They do not apply to the classical PVA/budget impact schemes, which have been discussed earlier.

Situations where risk sharing schemes could be considered by payers in the future include where:

• the objectives and scope of the scheme are explicit and transparent

• the new drug is a novel treatment in a high priority disease area with expected net health gain, and there are currently few if any effective treatments. In addition, translational science suggests good effectiveness in reality and delaying treatment before all the outcome data is available may not be in key stakeholder's interest. This can subsequently be verified by independent studies

• new drugs are seen as effective in priority disease areas but there are potential concerns with long term safety

• new drugs could have a substantial beneficial impact on service delivery and patient safety in practice but it has been difficult to prove this within the confines of a phase III trial

• the likely health gain can be determined within a limited time frame. This is especially important in patients with advanced cancers in order not to waste time and resources

• proposed patient access schemes in priority disease areas substantially lower health service costs to enhance reimbursement having factored in all administrative costs. This must though take into account the possibility of rebated drugs going out-of-date before they can be used or whether current systems have the ability to fully track patients and potential rebates, e.g. the cost of missed claims with bortezomid (above). Overall, rebate schemes do appear to provide a more accurate record of drug usage than the provision of free drug, as well as help ensure only appropriate patients are prescribed the new drug, enhancing their appeal

We believe proposed risk sharing schemes should be rejected where:

• effective and low cost standards already exist for the population in question with proven long term outcomes. This includes provisional reimbursement schemes which may encourage companies to launch new expensive products with only limited surrogate data

• Health Authorities will end up funding a substantial proportion of a new drug's development costs

• patient compliance is a key consideration to improve health, and this has not been adequately addressed in the proposed scheme

• there is a high administrative burden for the health service versus the likely health gain and/or perceived financial benefits

In addition, proposed schemes must be based on robust evidence ('coverage with evidence') for potential consideration. This includes robust translational science which has suggested beneficial outcomes in reality. Schemes must also include unambiguous and easily measured 'evidence based' effectiveness criteria evolved from good biological research and comprehensive clinical trials. Where there is insufficient evidence to make robust decisions, we believe reimbursement or funding should initially be considered only at similar prices to current standards. Prices can subsequently be increased in all or sub-populations as more data becomes available demonstrating increased value in practice. In this way limiting the financial exposure of payers as well as limiting Type 1 errors.

We believe key considerations when payers are reviewing proposed schemes include transparency, ethical considerations, staffing, evaluation and exit strategies. Transparency and ethical considerations include:

• Funding arrangements for any registries as well as administration costs must be transparent

• Any ethical, legal and clinical governance considerations must be fully addressed when proposing and developing future schemes. This includes issues of ownership of the data, especially if schemes are operated within health services, intellectual property rights and opportunities for appeal

• Future risk sharing schemes should be open to all pertinent companies and not just selected companies. This builds on the established procurement processes, and could be in the form of 'requests for risk sharing' schemes among competing companies in given disease areas/patient populations

Staffing/funding considerations include whether:

• Appropriate professionals are in place to fully evaluate proposed schemes, otherwise decisions may be compromised. This includes clinical, clinical pharmacology, pharmacy, IT and economic experts

• Continued funding of competent personnel (clinical and IT), as well as comprehensive IT infrastructures, is in place to effectively develop and implement follow-up registries where pertinent

• There are high ethical standards in the evaluation of proposed schemes. This includes the declaration of any contacts and conflicts of interest between experts and pharmaceutical companies that could potentially jeopardise evaluations

Finally, we believe evaluations must be built into future schemes based on good science given the paucity of published studies to date and concerns with many current schemes. In the past, pharmaceutical companies may have been unwilling to broadcast their schemes especially patient access schemes. However, there are now established payer networks to address this, and publication is seen as a major way forward for health authorities to learn from each another. Any evaluation must include the overall costs involved with implementing and conducting the schemes as well as their outcome. 'Exit' strategies must also be considered in advance should the effectiveness and/or safety of new drugs turn out to be worse in reality leading to their possible withdrawal during the lifetime of the scheme.

Consequently we believe future schemes must have realistic time scales, must not involve appreciable administrative burden if part of routine clinical care unless addressed, and must take cognisance of any likely changes in care during their lifetime, i.e. standard drugs losing their patent and/or clinical standards changing.

We hope that this paper will stimulate further debate and discussion especially in Europe about future risk sharing arrangements for pharmaceuticals amongst all key stakeholder groups.

## Competing interests

There are no conflicts of interest of any author. However, the majority of authors are employed by health authorities or health insurance agencies.

No author received financial assistance with writing this paper.

## Authors' contributions

JA - involved with appraising risk sharing schemes from across Europe and categorising them to provide direction to the Ministry of Health in Poland whilst developing new laws surrounding such schemes. KW and GOS provided support with this whilst both at the National Health Fund in Poland, and critiqued the final draft. BG - main author involved with constructing the general direction of the paper, collating the various schemes as well as producing the drafts including current concerns and future proposals for comment. BO - helped critique the discussions on Poland as well as the potential future direction. HH - critiqued the comments on Germany and provided guidance on the nature and construction of future schemes from a Physician Association perspective. OL - provided advice and comment on current schemes in Estonia and potential ways forward. SJ - actively involved with critiquing clinical and economic issues for new and existing drugs in HBCBS of NJ. This includes potential 'risk sharing' arrangements as well as programmes to enhance compliance, which is also seen as a growing problem especially for patients with chronic diseases. As a result, provided input and direction on the situation in USA. CS - provided feedback on current schemes in France. CZ - critiqued the recommendations based on her perspective as a payer in a leading region in Spain. MK - provided data on current schemes in Serbia as well as critiqued the future guidance based on her experience working for the Republic Institute for Health Insurance in Serbia. RG - actively involved in the first risk sharing schemes for Stockholm. Provided a different perspective when reviewing such schemes to give guidance for the future. KG - provided feedback on current schemes in Lithuania as well as critiqued proposed recommendations. AH - gave advice on the VELCADE scheme in the UK. He is also a member of a NICE appraisal group, and his knowledge was used to develop and critique proposed developments. SG - helped to critique the drafts based on his considerable experience in Italy and across Europe. LLG - contributed significantly to the discussions and future direction based on his extensive experience in Sweden with chairmanship of the Regional Drugs and Therapeutics Committee in Stockholm County Council, including methodologies to enhance the rational use of drugs, as well as general experience with informatics

All authors have read and approved the final manuscript

## Pre-publication history

The pre-publication history for this paper can be accessed here:

http://www.biomedcentral.com/1472-6963/10/153/prepub
